# Comparison of clinical findings in 293 dogs with suspect acute pancreatitis: Different clinical presentation with left lobe, right lobe or diffuse involvement of the pancreas

**DOI:** 10.4102/jsava.v91i0.2022

**Published:** 2020-04-21

**Authors:** Chad F. Berman, Remo G. Lobetti, Eric Lindquist

**Affiliations:** 1 Bryanston Veterinary Hospital, Johannesburg, South Africa; 2 Department of Companion Animal and Clinical Studies, Onderstepoort, South Africa; 3 Sonopath, New Jersey, United States

**Keywords:** gastroenterology, pancreatitis, internal medicine, companion animals, organ of nausea

## Abstract

Pancreatitis is a common clinical condition seen in companion animals. The correlation of the region of the pancreas affected to the presentation of clinical signs has not been previously described. A retrospective study on the clinical findings in 293 client-owned dogs diagnosed with suspect pancreatitis based on history, clinical signs, laboratory testing and abdominal ultrasonography was performed. Based on ultrasonography, dogs were divided into three groups: group 1: 41 dogs with ultrasonographic changes consistent with pancreatitis within the left lobe of the pancreas; group 2: 105 dogs with ultrasonographic changes compatible with pancreatitis within the right lobe of the pancreas; and group 3: 147 dogs with ultrasonographic evidence of diffuse pancreatitis. No significant differences regarding age, breed and sex were evident. Furthermore, statistical significance was demonstrated with the presence of pain in group 3; poor appetite in groups 2 and 3; and vomiting and diarrhoea in group 3. Pain is expected to occur with a higher frequency in diffuse pancreatitis, but it is not a common clinical sign. This may represent a more severe form of the disease when the pancreas is diffusely affected. Vomiting was more common than diarrhoea with both clinical signs more prevalent in dogs with diffuse pancreatitis, and this could be ascribed to gastric and intestinal tract involvement. Poor appetite occurred more frequently in dogs with diffuse and right lobe pancreatitis. A possible explanation can be attributed to the fact that the duodenum has many receptors and is referred to as the ‘organ of nausea’.

## Introduction

Pancreatitis is the most common condition of the exocrine pancreas in the dog and is defined as inflammation of the exocrine pancreas. This term includes diseases of the pancreas characterised by necrosis as well as irreversible structural changes such as fibrosis (Xenoulis 2015). Pancreatitis is generally divided into acute and chronic forms based on the absence or presence of certain histopathological features such as fibrosis and/or atrophy (Xenoulis, Suchodolski & Steiner 2008). The presence of permanent histopathological changes (namely fibrosis and acinar atrophy) is considered suggestive of chronic pancreatitis, whereas the absence of such changes together with an inflammatory reaction is more indicative of acute pancreatitis (Bostrom et al. 2013; Newman et al. 2004; Watson et al. 2007). Cellular infiltrates can also be used to differentiate pancreatitis into acute and chronic forms with suppurative inflammation or lymphocytic infiltration compatible with acute disease and chronic disease, respectively (Ferreri et al. 2003; Hill & Van Winkle 1993). However, histopathological differentiation is not always clear as many animals can have histopathological evidence of both acute and chronic pancreatitis (Xenoulis 2015).

The majority of dogs with pancreatitis are middle aged to old (usually > 5 years of age), but can vary from a few months to older than 15 years (Akol et al. 1993; Cook et al. 1993; Hess et al. 1998). The miniature schnauzer and terrier breeds are at an increased risk (De Cock et al. 2007; Ferreri et al. 2003). Most cases of pancreatitis are considered idiopathic although pathological conditions such as hypertriglyceridaemia, endocrine disease, adverse drug reactions, anti-convulsant therapy, surgery, and infectious and dietary factors have all been implicated (Xenoulis 2015). Although there are no pathognomonic clinical signs for pancreatitis in dogs, the typical presenting sign in dogs with acute severe pancreatitis is the acute onset of abdominal pain (Hess et al. 1998; Weatherton & Streeter 2009). Depending on disease severity, clinical presentation can vary markedly and may consist of non-specific findings such as poor appetite, vomiting, lethargy, diarrhoea, abdominal pain and weight loss. These are, however, non-specific clinical signs that can be seen with other conditions (Hess et al. 1998; Steiner 2003). The differential diagnostic list includes a plethora of primary diseases of the gastrointestinal, hepatobiliary and urogenital tract, intra-abdominal tumours, splenic torsion and hypoadrenocorticism. Dogs may also display a combination of different clinical signs, including dehydration, icterus, fever, hypothermia, bleeding diathesis or ascites (Hess et al. 1998), as well as present with severe systemic complications (Weatherton & Streeter 2009). These systemic complications include those already listed as well as tachycardia, arrhythmias, hypovolemic shock, acute respiratory distress syndrome and death.

The diagnosis of acute pancreatitis can be difficult because of the anatomic inaccessibility of the pancreas and vague clinical signs and findings on clinical examination. Despite numerous improvements in various diagnostic tests, the diagnosis of pancreatitis is still challenging. The only definitive diagnosis of pancreatitis is histopathology, which is highly invasive with localised disease possibly being missed with a single biopsy (Newman et al. 2004). Although routine clinical pathology is non-specific, it may help in estimating the severity of the pancreatitis (Xenoulis 2015). Animals with pancreatitis can show varying haematological abnormalities (Akol et al. 1993; Ferreri et al. 2003; Hess et al. 1998; Hill & Van Winkle 1993 ). Serum-specific lipase (cPL) has been shown to be both a sensitive and specific serum marker for pancreatitis in dogs (McCord et al. 2012; Steiner et al. 2008; Trivedi et al. 2011; Watson et al. 2010), with a sensitivity ranging between 72% and 78% (McCord et al. 2012) and a specificity of between 81% and 100% (Mansfield & Jones 2000; McCord et al. 2012; Neilson-Carley et al. 2011; Strombeck, Farver & Kaneko 1981; Trivedi et al. 2011). SNAP canine pancreatic lipase (cPL) has a sensitivity between 91% and 94% and a specificity between 71% and 78% for pancreatitis (McCord et al. 2012). Other diagnostic tests available include trypsin-like immunoreactivity (TLI), serum lipase and amylase activity, triolein and 1,2-*o*-dilauryl-rac-glycero-3-glutaric acid-6-methylresorufin, which is a lipase-based test (DGGR lipase assay, precision pancreatic specific lipase [PSL]). Recent studies have shown a high agreement between the Spec cPL and the DGGR lipase assay. However, this same article did demonstrate a fair agreement between pancreatic ultrasonography results and serum lipase results (Kook et al. 2014). Thus, lipase results are more accurate, and so ultrasonography should therefore be interpreted carefully. In the dog radiography is an insensitive diagnostic modality for pancreatitis because of non-specific findings and often no findings at all. It does, however, aid in ruling out other possible differentials (Akol et al. 1993; Hess et al. 1998; Hill & Van Winkle 1993). Contrast-enhanced computed tomography (CT) was reported to be promising in the diagnosis of two cases of canine pancreatitis (Jaeger et al. 2003); however, another study reported a low sensitivity for diagnosing pancreatitis in a small number of cases (*n* = 7) (Shanaman et al. 2013). Moreover, a pilot study of CT angiography in 10 dogs allowed a more complete evaluation of the entire pancreas than did ultrasound. In addition to this, identification of a heterogeneous contrast enhancement of the pancreas may be a negative prognostic indicator in dogs with acute necrotising pancreatitis (Adrian et al. 2015). Furthermore, in humans, contrast CT is frequently used for the diagnosis of pancreatitis (Arvanitakis et al. 2007; Cappell 2008; Kim & Pickhardt 2007; Scaglione et al. 2008; Sheu et al. 2012). To the authors’ knowledge, the use of magnetic resonance imaging (MRI) to investigate the canine pancreas has not been reported in the literature.

Ultrasonography is an alternative technique for imaging the pancreas and is considered the imaging method of choice for the diagnosis of pancreatitis in dogs (Xenoulis 2015), with a reported sensitivity of approximately 68% in dogs with acute severe pancreatitis; however, this is operator dependant (Hess et al. 1998; Ferreri et al. 2003; Saunders et al. 2002; Swift et al. 2000). The specificity of this modality has, to date, not been reported as no histopathology confirmation has been performed to establish this (Mansfield 2012). Contrast-enhanced ultrasound has been used in dogs with pancreatitis and can demonstrate pancreatic perfusion changes. It has also been shown to aid in the diagnosis of pancreatitis, pancreatic necrosis as well as disease monitoring in dogs following therapy (Lim et al. 2015; Rademacher et al. 2016). Thus, the clinical diagnosis of pancreatitis is generally based on a combination of clinicopathologic and imaging findings.

The aims of this study were to compare the clinical signs with the ultrasonographic findings in dogs with acute pancreatitis and to account for possible differences in the clinical presentation depending on the region of the pancreas affected as determined by ultrasonography.

## Materials and methods

Multi-institutional cross-sectional retrospective study was performed from first opinion and referral practices in Canada, North America and South Africa. Medical records were searched in which a final diagnosis of pancreatitis had been made based on typical history and clinical signs, laboratory testing and abdominal ultrasonography over a 24-month period (2013–2014). A total of 293 cases met the search criteria.

**FIGURE 1:**
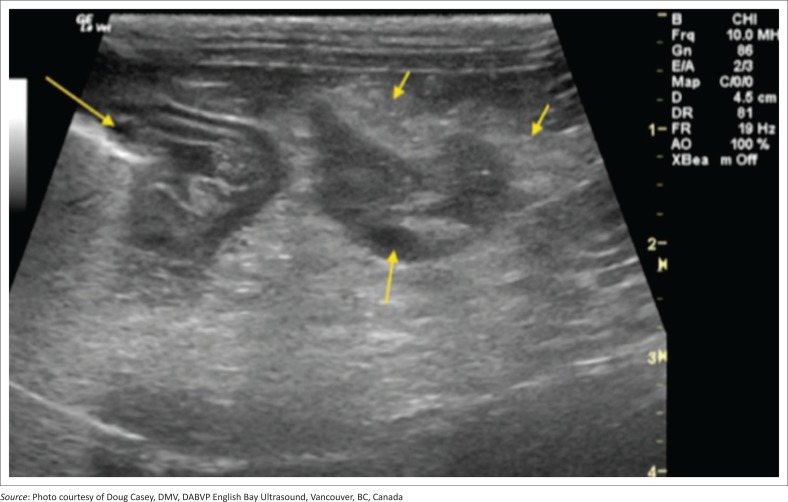
Example of pancreatitis affecting the left limb of the pancreas (group 1): Focal mixed hypoechoic lesion (middle arrow) in the near field at the base of the left pancreatic limb. Low-grade ill-defined inflamed fat (small arrows) is noted bordering the hypoechoic pancreatic parenchyma typical of pancreatitis. The location is caudal to the gastric fundus (long arrow) free from influence upon the duodenum or pyloric outflow.

**FIGURE 2:**
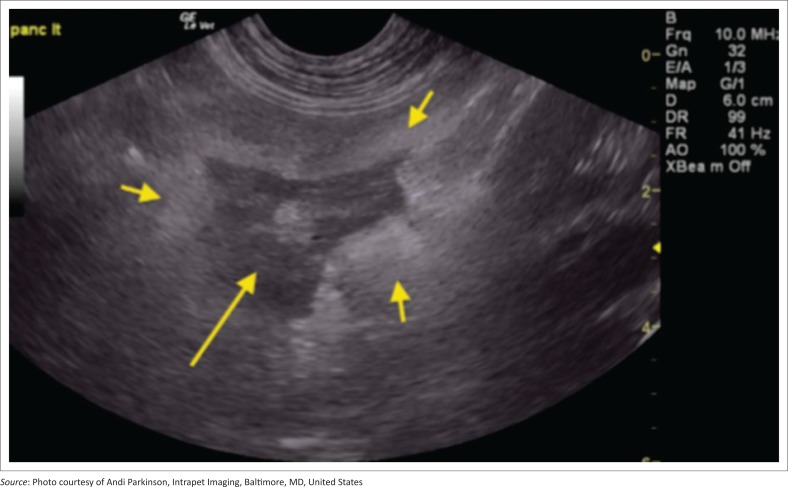
Example of pancreatitis affecting the left limb of the pancreas (group 1): Left limb of the pancreas demonstrating coarse mixed hypoechoic (long arrow) pancreatic parenchyma with ill-defined hyperechoic surrounding fat (small arrows) typical of acute pancreatitis.

**FIGURE 3:**
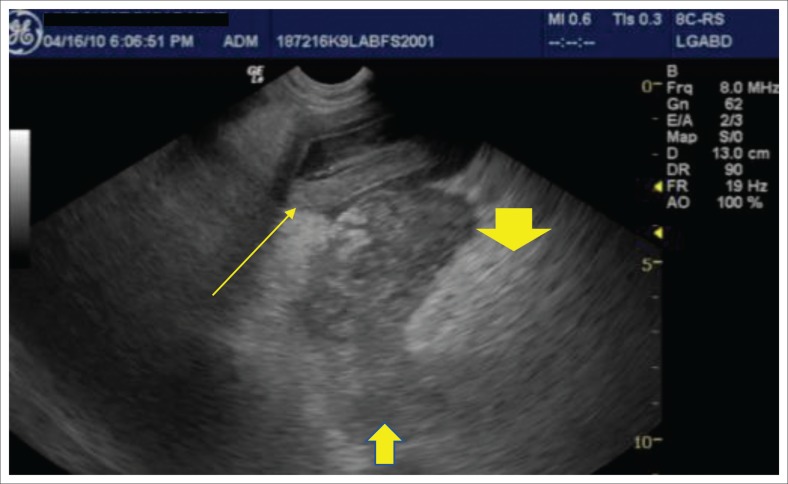
Example of pancreatitis affecting the right limb of the pancreas (group 2): Hypoechoic oedematous pancreatic parenchyma (middle arrow) and hyperechoic ill-defined surrounding fat (small thick arrow) consistent with saponification and inflamed mesentery. The upper descending duodenum in the near field is mildly oedematous and adjacent to the pancreatic inflammation (long thin arrow).

**FIGURE 4:**
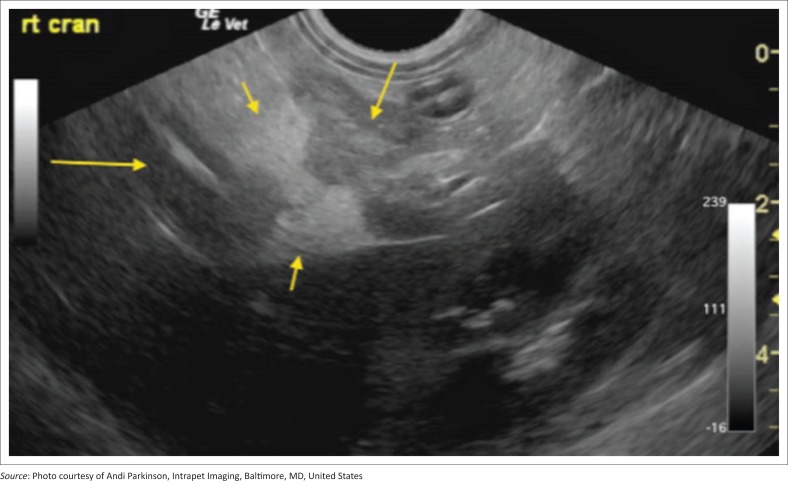
Example of pancreatitis affecting the right limb of the pancreas (group 2): Right limb pancreatitis with mixed hypoechoic oedematous parenchyma (middle arrow), ill-defined inflamed surrounding fat (short arrows), all of which surround promote inflammation of the upper descending duodenum (long arrow). Right kidney is present in far field.

Inclusion criteria into the study included supportive clinical signs and/or supportive clinical examination findings, supportive ultrasonographic findings as well as an abnormal SNAP cPL test. Animals had to have both appropriate ultrasonographic findings and an abnormal SNAP cPL to be included as well as ≥ 1 clinical sign and/or clinical examination finding. Indicative presenting clinical signs were the acute (< 2 weeks) onset of poor appetite, and/or vomiting or diarrhoea. Suggestive clinical examination findings were abdominal discomfort and/or pain on palpation. Full abdominal cavity ultrasound was performed by using an ultrasound machine (General Electric Logic E Ultrasound machine, GE Healthcare Biosciences, Shenzhen-China) with an 8-MHz probe, and the same type of machine and probe were used across multiple institutions. Findings consistent with pancreatitis are an abnormal echogenic appearance of the pancreas, which included a mixed hypoechoic pancreatic parenchyma and irregular capsule, and the presence of ill-defined hyperechoic surrounding peri-pancreatic fat and mesentery (Figures 1–4). In addition to this, assessment for the dilatation of the pancreatic or biliary ducts as well as the presence of any ascites was performed (Hecht & Henry 2007). Moreover, ultrasonographic changes (as described above) had to be restricted to the left or right lobe of the pancreas exclusively for patients to be classified as left- or right-sided pancreatitis. In addition, any involvement of the body of the pancreas would be ascribed as diffuse pancreatitis, and any dilatation of the pancreatic or biliary duct would be assigned to the right lobe pancreatitis group. In all cases the left, right and body of the pancreatic lobes were available for review. Unobstructed images of the left, right and body of the pancreas as well as a full video sweep of all areas of the pancreas were required to be included into the study. Multiple sonographers with training in small animal ultrasound performed the initial ultrasound scans. All ultrasonographers were either board certified internal medicine specialists or clinical sonographers and had to follow a standardised ultrasound procedure taking images and videos of all appropriate organs, reducing variability amongst the different sonographers. All ultrasound images and videos were subsequently reviewed in a blind manner by one author (E.L.) with extensive experience in clinical sonography (Diplomate of the American Board of Veterinary Practitioners [DABVP], certified International Veterinary Ultrasound Society [Cert.IVUSS]). Exclusion criterion was the presence of other primary diseases (such as gastrointestinal, hepato-biliary, urinary tract diseases) and incomplete visualisation of the pancreas on ultrasound. Animals with no ultrasonographic changes to the pancreas were also excluded from this study. Based on ultrasonographic findings, dogs were divided into three groups: group 1 – 41 dogs with changes within the left lobe of the pancreas exclusively; group 2 – 105 dogs with changes within the right lobe of the pancreas exclusively; and group 3 – 147 dogs with diffuse pancreatic involvement.

As this was a retrospective study, abdominal pain was either present or absent and not graded according to a pain scale. Each animal had their entire abdomen palpated from cranial to caudal and dorsal to ventral. If any discomfort or vocalisation was demonstrated, pain was regarded as present. In addition, if no discomfort and/or vocalisation was exhibited, pain was interpreted as absent. Poor appetite, vomiting and diarrhoea were recorded as either present or absent. Any dog with either complete loss or reduced appetite up to and including 2 weeks prior to presentation as described by the owner would be assigned the clinical sign of poor appetite.

All data were tabulated in an Excel spreadsheet programme (Excel^®^, Microsoft Corporation, Washington, United States.) and statistical analysis was performed by using a statistical software package (NCSS^®^, Kaysville, UT, United States). Descriptive statistics were used to describe the data. For single parameters (abdominal pain, poor appetite, vomiting and diarrhoea), differences between the groups were tested using one-way analysis of variance with Bonferroni and Tukey–Kramer comparisons. Fischer’s exact test was used to determine the association between ultrasonographic findings and clinical signs. The data were normally distributed, and the level of significance was set at *p* < 0.05.

### Ethical considerations

This article followed all ethical standards for carrying out a research without direct contact with human or animal subjects. No ethical approval was required as this was a retrospective non-invasive clinical study.

## Results

A total of 293 client-owned dogs were used with a median age of the groups 1–3 were 8.8, 9.8 and 10.1 years, respectively, with no statistical difference between the groups. Various breeds were present with no one breed or sex appearing to be over-represented in any one group. The following breeds were represented in the order of decreasing frequency: mixed breeds (128), Yorkshire terrier/Yorkshire terrier cross (31), Shih Tzu (18), Maltese/Maltese cross (18), Labrador/Labrador mix (17), Bischon Frise (16), Miniature Schnauzer (11), Dachshund (7), Terrier/Terrier mix (4), Boxer (3), Golden Retriever/Golden Retriever mix (3), Miniature Poodle (3), two of each American Eskimo mix, Bassett Hound, Beagle, Border Collie/Border Collie cross, Chihuahua/Chihuahua mix, Husky mix, Jack Russell terrier/Jack Russel terrier mix, Pomeranian, Shetland Sheepdog and Weimaraner; and one of each Akita, Boston Terrier, Cocker Spaniel, Collie mix, English Setter, Fox terrier, Lhasa Apso mix, Miniature Pincher, Pekingese, Pitbull mix, Pyrenees Mountain dog, Rat terrier, Vizsla and West Highland White terrier. Sex distribution in group 1 was 18 males and 23 females; in group 2, it was 52 males and 53 females; and in group 3, it was 68 males and 79 females, with no statistical difference between the groups.

**FIGURE 5:**
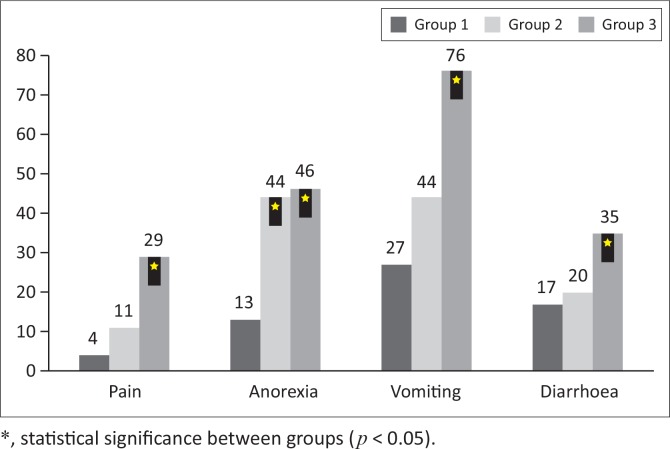
Clinical signs between the three groups with number of dogs exhibiting the signs. Group 1 – pancreatitis of the left lobe, group 2 – pancreatitis within the right lobe and group 3 – diffuse pancreatitis.

In group 1, pain was noted in 4/41 dogs (10%), poor appetite in 13/41 dogs (32%), vomiting in 27/41 dogs (66%) and diarrhoea in 17/41 dogs (41%). In group 2, pain was present in 11/105 dogs (10%), poor appetite in 44/105 dogs (42%), vomiting in 44/105 dogs (42%) and diarrhoea in 20/105 dogs (19%). In group 3, pain was noted in 29/147 dogs (20%), poor appetite in 46/147 dogs (31%), vomiting in 76/147 dogs (52%) and diarrhoea in 35/147 dogs (24%) (Table 1). Statistical significance was presence of pain in group 3; poor appetite in groups 2 and 3; and vomiting and diarrhoea in group 3 (Figure 5).

**TABLE 1:** Percentage of dogs displaying clinical signs according to the region of pancreas affected.

Group	Clinical signs (% dogs affected)			
	Pain	Anorexia	Vomiting	Diarrhoea
1 (left limb)	11	32	66	41
2 (right limb)	10	42	42	19
3 (diffuse)	20	31	52	24

## Discussion

This study focused on relating clinical signs of pancreatitis to the area of the pancreas affected, and it documented different presenting clinical signs depending on which lobe of the pancreas was involved. No obvious breed predisposition was identified in this study, which agrees with previous studies that reported that dogs of any age, breed or sex can develop pancreatitis. Most dogs that are presented with pancreatitis are usually greater than 5 years of age (Cook et al. 1993; Ferreri et al. 2003; Hess et al. 1998; Watson et al. 2010). Various studies have highlighted differences in breed predilections because of different geographic regions. In the United States, miniature schnauzers and terrier breeds (particularly Yorkshire terriers) are at increased risk (Cook et al. 1993; Hess et al. 1998; Lem et al. 2008). In the United Kingdom, Cocker spaniels, Cavalier King Charles spaniels, Border collies and Boxers have been reported to be at increased risk for chronic pancreatitis (Watson et al. 2007).

The differences between this current study and those previously reported can be ascribed to the difference in population size, where sample size in the current study was significantly higher than that in previous studies.

In the dog, the pancreas is divided into the right and left lobes, joined at the body with the right lobe easiest to identify on ultrasound, located dorsomedial to the descending duodenum, ventral to the right kidney and lateral to the ascending colon (Larson 2016). The duodenum and right pancreatic lobe can be located in the dorsal aspect of the 10th–12th intercostal space (can vary with dog conformation) and followed caudally, either in transverse or longitudinal plane (Larson 2016). The pancreatic body lies immediately caudal and dorsal to the pyloroduodenal junction, ventral to the portal vein and medial to the proximal duodenum. The left lobe in the dog lies dorsal and caudal to the body of the stomach, cranial to the transverse colon. It follows the course of the splenic vein as it travels from the splenic hilus medially to the portal vein at the level of the pancreatic body. The canine left lobe is often much smaller than the right lobe and is more difficult to consistently detect because of shadowing and reverberation artefact from gas in the stomach and transverse colon (Larson 2016). The anatomic location of each lobe is important as it can explain the findings of this study.

Abdominal ultrasound is considered the imaging method of choice for the diagnosis of pancreatitis in dogs with the added advantage of ruling out differential diagnoses that share similar clinical signs (Xenoulis 2015). The performance of abdominal ultrasonography is, however, dependent on the expertise of the user. Ultrasonographic findings in dogs with acute pancreatitis include hypoechoic areas within the pancreas, increased echogenicity of the mesentery and enlargement or irregularity of the pancreas as well as dilatation of the pancreatic or biliary duct, and ascites (Ferreri et al. 2003; Hecht & Henry 2007; Hess et al. 1998; Saunders et al. 2002; Swift et al. 2000). Dilatation of the pancreatic duct is considered pathognomonic for pancreatitis in human patients, and possibly also in dogs (Simpson & Lamb 1995; Watson 2004). In pancreatitis, the surrounding mesentery can be hyperechoic indicating peri-pancreatic steatitis and fat necrosis (Hecht & Henry 2007). In the dog, the right lobe seems to be the most commonly affected portion of the pancreas in acute disease, which may be related to the fact that the right limb is more easily identified on ultrasound (Larson 2016). These ultrasonographic findings were present in this current study and were used to aid the researchers in the diagnosis of acute pancreatitis. Previous research has described normal ultrasonographic findings in healthy dogs. They showed that the right lobe of the pancreas was most consistently visible and measured in 98% of dogs versus the left lobe at 88% of all dogs and the body at 64%, with the pancreatic duct detected in only 8% of all the dogs (Murtaugh et al. 1985). In an experimental study of induced pancreatitis in six healthy dogs, an ill-defined mass with a heterogeneous echogenicity accompanied by an overall decrease in echogenicity was reported in both the left and right pancreatic lobes. Right pancreatic lobe involvement was detected in 5/6 dogs, and the left pancreatic lobe was involved in 4/6 dogs (Murtaugh et al. 1985). In a study that assessed the ultrasonographic findings in three dogs with experimentally induced pancreatitis, right pancreatic lobe pathology was detected more often than the left lobe pathology (Nyland et al. 1983). These findings are also in agreement with another study (Lamb & Simpson 1995). Although these researches are experimental and were conducted on a small number of dogs, these results are, however, like the findings in our study where lesions isolated to the right lobe of the pancreas was identified in 36% of cases in comparison with lesions isolated to the left lobe of the pancreas in 14% of cases. Diffuse pancreatic disease was detected in 50% of patients. Thus, based on our study, diffuse pancreatic pathology seems to be the most common ultrasonographic finding in patients with acute pancreatitis.

Ultrasonographic findings consistent with acute pancreatitis were noted in 68% of cases in a previous study (Hess et al. 1998). Ultrasonography has advantages over other imaging methods in being relatively low in cost and non-invasive. However, the low negative predictive value of normal ultrasonographic findings prevents the use of ultrasonography as an exclusion diagnostic procedure for acute pancreatitis as false-negative results are possible (Ruaux 2003). Not all lesions can be correlated with the clinical status of the patient (Mix & Jones 2006). As non-significant pancreatic lesions can be detected on ultrasound examination, the significance of pancreatic abnormalities identified via ultrasonography must be interpreted in the light of a patient’s clinical signs (Mix & Jones 2006). A previous study showed that the ultrasound pattern of corrugated and thickened bowel wall was associated with pancreatitis. In that study 18 dogs had a corrugated bowel; however, a final diagnosis of acute pancreatitis was made in only 50% of cases (Moon, Biller & Armbrust 2003). Therefore, a diagnosis of pancreatitis should not be based exclusively on ultrasonographic findings, and correlating appropriate history, clinical examination findings and biochemical blood tests are still crucial.

No clinical sign is pathognomonic for pancreatitis in dogs; however, dogs with severe acute pancreatitis are typically presented with acute onset abdominal pain (Hess et al. 1998; Weatherton & Streeter 2009). One study reported abdominal pain in 58% of dogs with acute pancreatitis (Hess et al. 1998), which is much higher than the findings in this current study where abdominal pain was only present in 15% of all cases. Poor appetite has been reported to occur in 91% of cases (Hess et al. 1998), which is different from the current study where a poor appetite was present in only 35% of all dogs in the study. Acute vomiting has been reported in 90% (Hess et al. 1998) of cases, whereas in this study it occurred in only 50% of all patients. Diarrhoea has been reported in 33% (Hess et al. 1998) of dogs, whereas in this study it occurred only in 25% of all animals. The differences between the current study and Hess et al.(1998) can be ascribed to the different focus of the two studies as well as improvement with abdominal ultrasonography technology and operator expertise. Furthermore, the sample size in the current study was significantly higher than that of Hess et al.’s study. Moreover, differences amongst these results could also be attributed to the dissimilar patient populations. Hess et al.’s (1998) study only contained patients that died; thus, those patients may have had more severe clinical signs. In another study on 20 dogs with pancreatitis, only one dog (5%) did not have gastrointestinal signs (Kis et al. 2013). Once again this was a very small sample size.

In addition to this, pain, vomiting and diarrhoea were more commonly identified in diffuse pancreatitis. Poor appetite was more prevalent in right-sided and diffuse pancreatitis. These differences between the groups can possibly be attributed to gastric and/or intestinal tract involvement when various lobes of the pancreas are affected. In humans, numerous studies have demonstrated the relationship of nausea with the onset of gastric dysrhythmias in individuals with motion sickness, pregnant women and gastroparesis (Hasler et al. 1995; Koch 2014; Xu et al. 1993). Previous studies have suggested a relationship between gastric dysrhythmias and nausea (Xu et al. 1993). In addition to this, medications that decrease dysrhythmias, decrease nausea and stimuli that increase dysrhythmias may promote the sensation of nausea (Koch 2014). It can thus be speculated that diffuse pancreatitis, because of its close association with the stomach, may result in gastric dysrhythmias that would cause nausea and vomiting. Possibilities for the increased frequency of diarrhoea in diffuse pancreatitis would be thickening of the gastric and colonic wall and/or gastric dumping syndrome. A recent study in humans with acute pancreatitis, by using MRI to record abnormalities in the gastrointestinal tract, showed a thickened stomach and transverse colon in 20% and 15% of patients, respectively (Ji et al. 2017). In humans following gastrectomy a dumping syndrome can develop. This entails rapid gastric emptying of hyperosmolar contents into the proximal intestine (Davis & Ripley 2017; Machella 1950). Because of the proximity of the left lobe of the pancreas with the stomach, it can be speculated that gastric motility may be affected because of the localised inflammation, resulting in dumping of gastric contents into the proximal intestine causing diarrhoea. In humans, an acute necrotising pancreatitis may result in colonic complications. A previous study demonstrated that in human patients with surgical management of acute necrotising pancreatitis, 6.1% had colonic infarction secondary to the inflammatory process as a complication (Adams, Davis & Anderson 1994). These findings are similar to a previous study which showed in 22 patients with acute pancreatitis, nine had colonic involvement. The transverse colon was affected in three of the nine patients (Aldridge et al. 1989). In the dog the left lobe of the pancreas lies cranially to the transverse colon. Albeit not as severe as the complications that develop in people, we hypothesise that the localised inflammatory process affecting the left lobe of the pancreas might result in a colitis of the transverse colon and may not necessarily always cause vomiting; however, this may change overtime as the condition progresses. Although mild clinical signs associated with left lobe pancreatitis have not been described in the veterinary literature, a new publication assessing CT angiography and ultrasonography in dogs with acute pancreatitis has demonstrated some interesting findings. This study showed that dogs with heterogeneous contrast enhancement of the pancreas had significantly longer duration of hospitalisation including the likelihood to be hospitalised for more than 5 days, had increased number of relapses and were significantly more likely to have portal vein thrombosis (French et al. 2019). A recent study highlighted that there was a weak but significant linear correlation between thickness of right lobe of the pancreas with that of the mural thickness of the duodenum and the duodenal diameter (Wickramasekara Rajapakshage et al. 2016). This demonstrates a dimensional relationship between the right pancreatic limb and adjacent duodenum and can be used to assess pancreatic size in dogs (Wickramasekara Rajapakshage et al. 2016). In addition, recent studies have demonstrated that the intestine is thought to contribute to or exacerbate pancreatic inflammation directly because of intestinal ischaemia (Flint & Windsor 2003). The right pancreatic lobe lies dorsomedial to the descending duodenum. Poor appetite was a statistical finding in this study, and it did appear more prevalent in dogs with right-sided and diffuse pancreatitis. A possible explanation can be ascribed to the fact that the duodenum has a large number of receptors and is referred to as the ‘organ of nausea’ (Schoor 2011). Stimulation of these receptors within the duodenum could have resulted in the poor appetite because of nausea and not necessarily vomiting. Furthermore, pain seen in canine patients with acute pancreatitis was noted with a significantly higher frequency in diffuse pancreatic disease compared with disease restricted to the left or right lobe of the pancreas. This may be because dogs with diffuse pancreatitis have a more severe form of the disease. Despite overlap between groups, this study indicates that pain response is expected to occur with a higher frequency in diffuse pancreatitis but overall is not a very common clinical sign. This is important as lack of abdominal pain does not exclude pancreatitis especially if either of the two lobes is affected.

The results of our study are in contrast to a previous study that showed no significant differences between clinical signs and ultrasonographic changes in the pancreas (Myung-Jin et al. 2017). However, that study had a small sample size of 40 client-owned dogs (Myung-Jin et al. 2017). In addition to this, the aims of that study focussed on comparing abnormal serum canine pancreas-specific lipase results and pancreatic ultrasonographic changes in dogs with pancreatitis, which are different from the aims of our study.

Important limitations of this study were as follows: it was a retrospective study with dogs assessed only at one single time point; only a single ultrasound examination was performed with no follow-up scans; and no pancreatic histopathology was conducted. Furthermore, multiple individuals performed the ultrasound, and thus there could have been interobserver variability as the skills of the operator as well as the sensitivity of the ultrasound scanner are extremely important with ultrasonography. However, all persons performing the ultrasonography had training in sonography and had to follow a standard ultrasonographic procedure using the same ultrasound scanner. Moreover, all images and videos were assessed by one reviewer with extensive experience in small animal ultrasound, which reduced the impact of these limitations. Although grading for lesions (clinical and ultrasonographic findings) observed in dogs with pancreatitis was not discussed, we do not believe that this influenced the outcome of our results as presence and not severity of clinical signs were the aims of our study. However, future studies assessing prognostic significance of these findings should include a grading scale for abdominal pain and poor appetite. Additional limitations were that the true prevalence of pancreatitis confined to the limb of the pancreas may have been under-represented in this study as interference from gastric content may have resulted in animals with left limb pancreatitis being unnecessarily excluded, and no odds ratio was performed to determine any breed predilection. Dogs had to have ultrasonographic findings consistent with pancreatitis to be included into the study. Thus, patients with no ultrasonographic findings may have been excluded from the study, which may have biased the results. However, it is unlikely that these patients would have had the other inclusion criteria. Moreover, the concentration of serum canine pancreatic lipase was not assessed. The definitive Spec cPLI concentration test has a grey zone when the result is between 200 mg/L and 400 mg/L and values > 400 mg/L indicate pancreatitis. The SNAP cPL results can only be differentiated as normal or abnormal. Thus, some of the patients may have had a false-positive test result for the SNAP cPL.

Despite the limitations of the study, the findings nevertheless present pertinent questions, including how dogs with pancreatitis may display a variety of different clinical signs depending on the region of the pancreas affected. Although these results have not been previously described, these findings are important, as they may aid in the understanding of how these vague clinical signs occur in the dog and may help practitioners with their treatment regimens. Moreover, further studies are required to validate these results and to assess whether the area of the pancreas affected has prognostic significance in the dog.

Based on the results obtained in this study, it can be concluded that animals with left, right and diffuse pancreatitis have different clinical presentations. In addition, pain occurs with a higher frequency in diffuse pancreatitis, although vomiting and diarrhoea are more prevalent in dogs with diffuse pancreatitis. Furthermore, reduced appetite appears more commonly in dogs with diffuse and right lobe pancreatitis.
